# Novel Indole–Thiazole Derivative Containing a *p*-Nitro Substituent (CS03): Validation of an HPLC-UV Quantification Method

**DOI:** 10.1021/acsomega.5c01148

**Published:** 2025-04-09

**Authors:** José Cleberson Santos Soares, Iago Dillion Lima Cavalcanti, Iranildo José da Cruz-Filho, Mariane Cajubá de Britto Lira Nogueira, Maria do Carmo Alves de Lima

**Affiliations:** 1 Laboratório de Química e Inovação Terapêutica (LQIT), Universidade Federal de Pernambuco (UFPE), Recife 50670-901, Brazil; 2 Instituto Keizo-Asami (iLIKA), Universidade Federal de Pernambuco (UFPE), Recife 50670-901, Brazil; 3 Laboratório de Nanotecnologia, Biotecnologia e Cultura de Células, Centro Acadêmico de Vitória, Universidade Federal de Pernambuco (CAV/UFPE), Recife 50670-901, Brazil

## Abstract

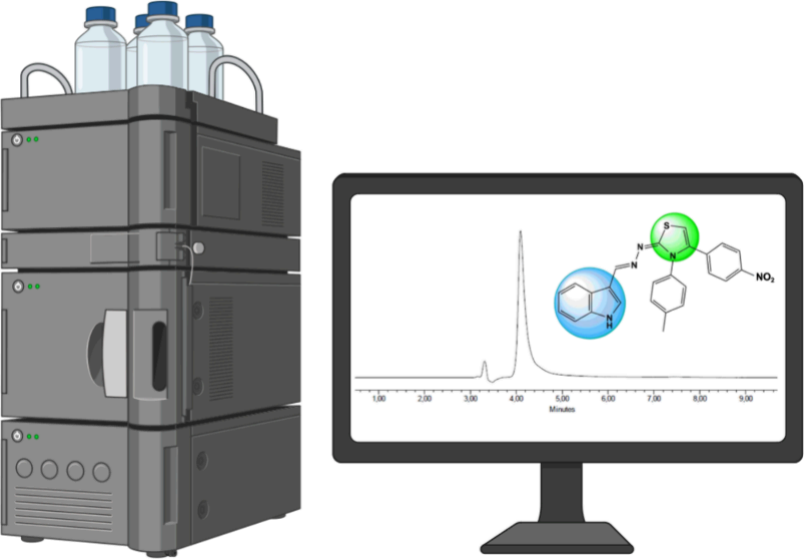

The validation of an analytical method enables the identification of the physicochemical characteristics of a molecule, aiding in the development of new drugs and allowing for its dosage in pharmaceutical forms. This is a crucial step in the creation of new pharmaceutical products. This article aims to validate a method for quantifying a novel indole–thiazole derivative with a *p*-nitro substituent (CS03) encapsulated in nanoparticles. The CS03 quantification method was evaluated using HPLC-UV techniques based on selectivity, linearity, accuracy, precision, detection and quantification limits, and robustness. Additionally, the stability of CS03 in various simulated pH environments and its encapsulation in polysaccharide-coated nanoparticles were assessed. The method proved effective in quantifying CS03, demonstrating selectivity, linearity, precision, and accuracy, with detection and quantification limits appropriate for measuring the molecule postencapsulation in nanoparticles. The validated method is suitable for determining CS03, facilitating studies focused on the clinical application of this molecule for new drug development.

## 1 Introduction

The combination of indole and thiazole nuclei has proven effective in developing new compounds with potential antitumor properties and low toxicity for normal cells.^1,2^ These findings highlight the significance of these nuclei and suggest the possibility of developing new molecules from their association, like CS03, an indole-thiazole derivative with a p-nitro substituent.^3,4^ Preliminary studies by Soares,^3^ Soares et al.,^4^ and Soares^5^ have shown that this molecule exhibits antiproliferative properties against breast cancer lines (MCF-7 and T-47D), prostate carcinoma (DU-145), and acute leukemia T cells (Jurkat). These results indicate that CS03 may be promising for pharmaceutical applications. However, it is necessary to develop a validated analytical method to evaluate the physicochemical characteristics of the molecule under various conditions, to better understand its behavior in future in vitro and/or in vivo studies.

The validation of an analytical method aims to demonstrate that the proposed method is suitable for quantifying an analyte in a matrix at a certain concentration level, with satisfactory accuracy and precision.^6,7^ The analytical method can identify the physicochemical stability of a drug candidate molecule in different biological conditions, as well as its solubility, bioavailability, and pharmacokinetic properties, which will allow for evaluating the compound’s behavior in the human body.^8−10^

To determine the physicochemical characteristics of CS03, the validation of a method is essential. This validation would also enable its encapsulation in nanosystems, allowing the identification of the encapsulation rate through a robust analytical method, such as High-performance liquid chromatography (HPLC) coupled with an ultraviolet (UV) detector.^11−13^ Encapsulation of a molecule in nanocarriers necessitates an analyte method that can determine when the compound was encapsulated in the nanocarrier, and the HPLC-UV method is a viable option as it allows the identification of an analyte in a matrix by separating the study analyte based on its specific wavelength.^7,14−16^ Polymeric nanoparticles facilitate the encapsulation of molecules with controlled release. Coating these nanosystems with polysaccharides such as dextran and fucoidan enables targeting specific sites and potentially enhancing the therapeutic effect of the encapsulated molecule due to additional pharmacological activities such as antitumor and anti-inflammatory effects.^17,18^

Based on the stringent parameters established by regulatory agencies such as the International Conference on Harmonization (ICH), the Food and Drug Administration (FDA), and the Brazilian Agency for Health Surveillance (ANVISA),^19−22^ the validation of an analytical method for the quantification of CS03 in different matrices has been proposed.

## 2 Materials and Methods

### 2.1 Materials

The polysaccharides (dextran and fucoidan, %purity ≥ 95) were purchased from Sigma-Aldrich (USA), as were the solvents acetonitrile (% purity 99.9% w/w, HPLC grade), trifluoracetic acid, and hydrochloric acid (HCl), as well as ethyl cyanoacrylate (ETCA), monobasic potassium phosphate, and dibasic potassium phosphate. The dialysis membrane was purchased from Spectra-por (Marne la Vall’ee, France) and Dimethyl sulfoxide (DMSO) P.A./ACS was obtained from NEON (São Paulo, Brazil).

### 2.2 Methods

#### 2.2.1 System Suitability Test

The parameters of number of theoretical plates, height of theoretical plates, tailing factor, retention time, and precision of injections were evaluated to assess the performance of the chromatographic method for quantifying CS03 in the system suitability test. To do this, 6 replicate injections of the CS03 solution (10 μg.mL^–1^) were used.

#### 2.2.2 Proposed Validation Method

**Figure 1:**
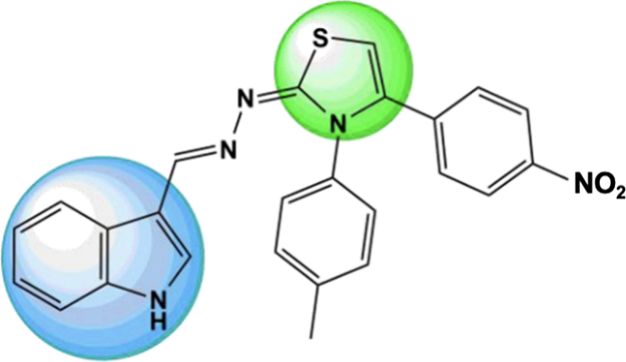
Chemical structure of CS03. Nuclei referring to the structure of indole (blue circle) and thiazole (green circle).

For the validation of the CS03 quantification method by HPLC (Waters Alliance 2695 Separation Module, Waters Corporation, Milford, USA) equipped with a 2489 UV–vis detector and 2998 PDA detector, the ICH,^19^ FDA,^20^ FDA,^21^ and ANVISA^22^ guidelines were adhered to. Good manufacturing practices were observed during the validation process, which was assessed through parameters including selectivity, linearity, accuracy, precision, limits of detection and quantification, and robustness. The method utilized a C18 column (3.5 μm 4.6 × 250 mm, XBridge) due to the hydrophobic nature of CS03 (Figure 1). The mobile phase consisted of acetonitrile:acidified water (0.05% trifluoroacetic acid, pH = 3) in an 85:15 v/v ratio, with an injection volume of 50 μL, a run time of 10 min, a flow rate of 0.80 mL.min^–1^, and a temperature of 25 °C. The wavelength for the UV detector was set at 348 nm for CS03.

##### 2.2.2.1 Selectivity

The selectivity of the analytical method for CS03 at a concentration of 10 μg.mL^–1^ was evaluated in a buffer solution simulating pH 7.4 for blood, pH 1.2 for gastric, and pH 6.8 for intestinal pH. The method was also used to quantify CS03 in the medium of the constituents of fucoidan or dextran nanoparticles. For this study, 5 replicates were carried out with independent runs of each matrix tested. The ability of the method to separate the peak of the molecule from the substances present in the matrix was evaluated based on the peak area and retention time.

##### 2.2.2.2 Linearity

A stock solution of CS03 (1 mg.mL^–1^) in DMSO:acetonitrile (1:1 v/v) was prepared and used for a maximum of 2 weeks, stored in a hermetically sealed amber bottle at −20 °C. The CS03 standard curve was obtained at concentrations of 1, 2.5, 5, 10, 15, and 20 μg.mL^–1^, prepared with the stock solution diluted in the mobile phase of the method under validation [acetonitrile:acidified water containing 0.05% trifluoroacetic acid, pH = 3, 85:15 (v/v)]. Linear least-squares regression was applied to fit the standard curves, weighted by the reciprocal of the concentration, with the peak area of CS03. The difference between the theoretical and experimental values of the standard curve allowed the residues to be identified.

##### 2.2.2.3 LOD and LOQ

The official ICH^19^ guidelines were used as a basis for obtaining the limit of detection (LOD) and limit of quantification (LOQ) of CS03 using the standard curve. The values of the standard deviation of the response and the slope of the standard curve were multiplied by 3.3 (Signal/Noise greater than or equal to 3.3) to obtain the LOD. The ratio between the standard deviation of the response and the slope of the CS03 standard curve multiplied by 10 (Signal/Noise greater than or equal to 10) was used to obtain the LOQ, which is defined as the smallest amount of the analyte that was quantified.

##### 2.2.2.4 Carryover

To evaluate the carry-over, an injection containing only the mobile phase of the method was injected into the HPLC-UV after injection of a concentration of 20 μg.mL^–1^ of CS03. The presence of a possible residue of CS03 was evaluated and calculated on the blank chromatogram. The test was performed six times.

##### 2.2.2.5 Accuracy

The accuracy of the HPLC-UV method of the CS03 was performed in triplicate using three different known concentrations of the molecule (1, 10, and 20 μg.mL^–1^) diluted in mobile phase. Recovery percentages were determined by calculating the ratio between observed and theoretical concentrations of CS03.

##### 2.2.2.6 Precision

Precision was assessed using CS03 solution (1, 10, and 20 μg.mL^–1^) diluted from the stock solution in acetonitrile:acidified water (0.05% trifluoroacetic acid, pH = 3) 85:15 (v/v) at 25 °C. Repeatability (intraday) and intermediate precision on separate days (interday) were evaluated, both by different analysts. For repeatability, fresh samples were used, prepared at the three concentrations determined, as for the precision interday, the evaluation took place by injecting the concentrations on different days. Three different analysts performed the injection of the CS03 samples at concentrations of 1, 10, and 20 μg.mL^–1^ to obtain the results. Relative standard deviation (%RSD) was used to express repeatability and intermediate precision results.

##### 2.2.2.7 Robustness

A 5% variation of the final conditions (low, medium, or high levels) of the chromatographic method under validation was considered for the robustness study. The parameters selected for variation were flow rate, column temperature, and mobile phase (Table 1).

**Table 1:** Variation of Analytical Parameters for the Quantification of CS03 in the HPLC-UV Method

**Parameter**	**Variation (Specification)**		
Concentration of acetonitrile in the mobile phase	80.75% (AA)	85% (Aa)	89.25% (aa)
Concentration of acidified water in the mobile phase	19.25% (BB)	15% (Bb)	10.75% (bb)
Column temperature	23.7 °C (CC)	25.0 °C (*Cc*)	26.3 °C (cc)
Mobile phase flow rate	0.76 (DD)	0.80 (Dd)	0.84 (dd)

In the robustness study, a total of 27 experimental runs were carried out on the HPLC-UV method with CS03 solution (10 μg.mL^–1^). Table S1 details the parameter combinations for each elution. The ratio between the observed and theoretical concentrations was used to determine the effects of the variables.

#### 2.2.3 Development of Nanoparticle Polysaccharide-Coated Containing CS03

For the development of nanoparticles, the anionic emulsion polymerization (AEP) method was followed.^17,23^ Briefly, 5 mg of the polysaccharide (fucoidan or dextran) was dissolved in 5 mL of ultrapure water and the pH of the solution was corrected to 2.5 using HCl (1 M). After that, the solution was stirred at 1200 rpm and the Ethyl cyanoacrylate (ETCA) monomer was added. After 2 min of ETCA addition, 500 μL of the CS03 solution (5 mg.mL^–1^) was added and the solution was kept under stirring for 3 h to ensure complete polymerization.

After the obtaining process, an aliquot of the dispersion was reserved for analysis of the CS03 content in the HPLC-UV. The dispersion was purified using a dialysis membrane (Spectra-por membrane 100 000 g/mol molecular weight cutoff (MWCO), Biovalley, Marne la Vall’ee, France) in 1L of distilled water with stirring at 300 rpm overnight. After purification, an aliquot of the dispersion was used to quantify the encapsulated CS03 and compare it with the value obtained from the content before dialysis.

## 3 Results and Discussion

### 3.1 System Suitability Test

The system suitability provides critical data regarding the compliance of chromatography, indicating the capability to reproduce a method with reliable outcomes and acceptable precision. As indicated by the results obtained for the CS03 quantification method in Table 2, the number of theoretical plates was 2,129 ± 164.61. According to the literature, a higher number of theoretical plates correlates with increased column efficiency. The theoretical plate height was 0.12 ± 0.01. According to studies, a lower plate height signifies a narrower bandwidth, thus enhancing column efficiency.^24^ The injection precision results conformed to the ICH^19^ guidelines, exhibiting an RSD value of less than 15%.

**Table 2:** System Suitability Data for the CS03 Quantification Method by HPLC-UV

**Parameters**	**Results**
Retention time (min)	4.21 ± 0.07
Area (average)	2,437,836 ± 76,739
Number of theoretical plates	2,129 ± 164.61
Theoretical plate height	0.12 ± 0.01
Tailing factor	1.45 ± 0.09
Injection precision (%RSD, *n* = 6)	3.15

The tailing factor indicates the symmetry of the chromatographic peak, and a value of between 1.2 and 1.5 is desirable.^24^ According to the results, a tail factor of 1.45 ± 0.09 was found. Given the results of the system’s suitability, the proposed method for quantifying CS03 by HPLC-UV appears to be suitable with the parameters selected in terms of column and mobile phase proportion.

### 3.2 Validation of the Method

The validation of the CS03 quantification method by HPLC-UV was determined from the stability analysis of the molecule in different simulated pHs and the encapsulation in two models of ETCA nanoparticles coated with dextran or fucoidan. The proposal was to understand the behavior of the molecule in future *in vivo* studies and the possibility of encapsulation of CS03 in nanoparticles to preserve its physicochemical characteristics.

The development of the analytical method for CS03 in HPLC-UV will allow evaluating of the behavior of this molecule in a complex pharmaceutical matrix, with high reproducibility and sensitivity, through a simple and fast technique.

#### 3.2.1 Selectivity

**Figure 2:**
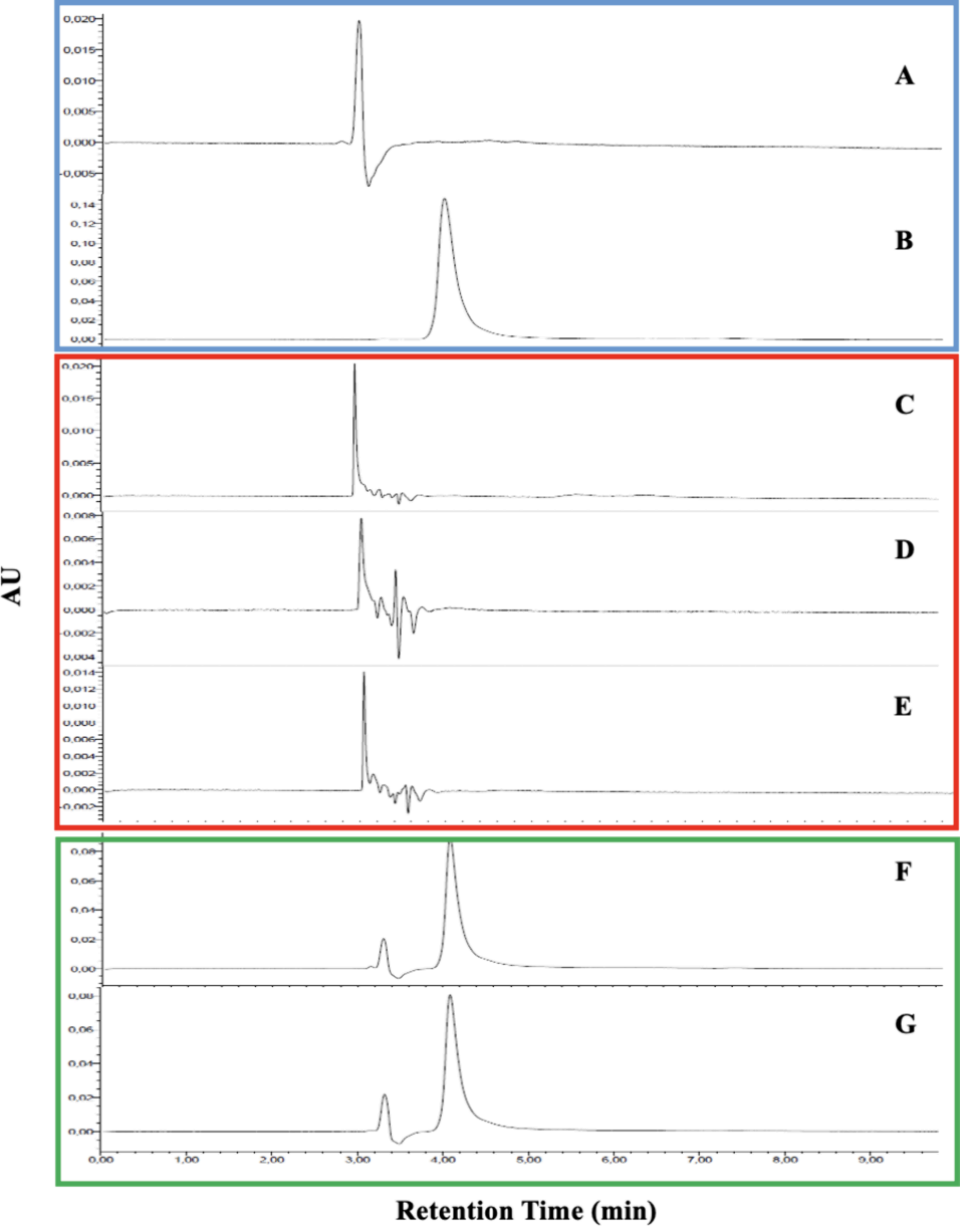
Chromatograms of the mobile phase (A) and CS03 (B) in the light blue box; CS03 in solutions of phosphate buffer pH 7.4 (C), gastric pH 1.2 (D), and intestinal pH 6.8 (E) in the red box; and CS03 in the medium of the nanoparticle constituent’s dextran (F) and fucoidan (G), in the green box.

The selectivity analysis of the method using reversed-phase chromatography with isocratic elution in a CS03 concentration of 10 μg.mL^–1^ in different pH solutions (pH= 7.6, 6.8, and 1.2) and in contact with the constituents of dextran or fucoidan nanoparticles, confirms that the method used allows the analysis of CS03. The chromatograms (Figure 2) show a good resolution of the CS03 peak, separating it from the other constituents of the matrix used. The peak retention time of CS03 was 4.21 ± 0.07 min.

The selectivity data obtained provides significant information, indicating that CS03 degrades in varying pH environments. Specifically, when attempting to quantify CS03 in simulated physiological pH solutions (7.4, 1.2, and 6.8) (Figure 1C–E, **red box**), it is observed that the CS03 peak shifts, accompanied by a reduction in peak intensity.^25,26^ These peaks likely represent degradation products of CS03, underscoring the molecule’s instability at pH levels of 1.2, 6.8, and 7.4, which may limit its potential therapeutic application in *in vivo* studies.

These findings highlight the necessity for developing pharmaceutical formulations that offer protection to the molecule when administered in the body.^27−29^ An example is omeprazole, which exhibits instability across a wide pH range, rapidly decomposing at pH levels below 7.8, thereby necessitating a specific pharmaceutical form for human administration.^27^

#### 3.2.2 Linearity

**Figure 3:**
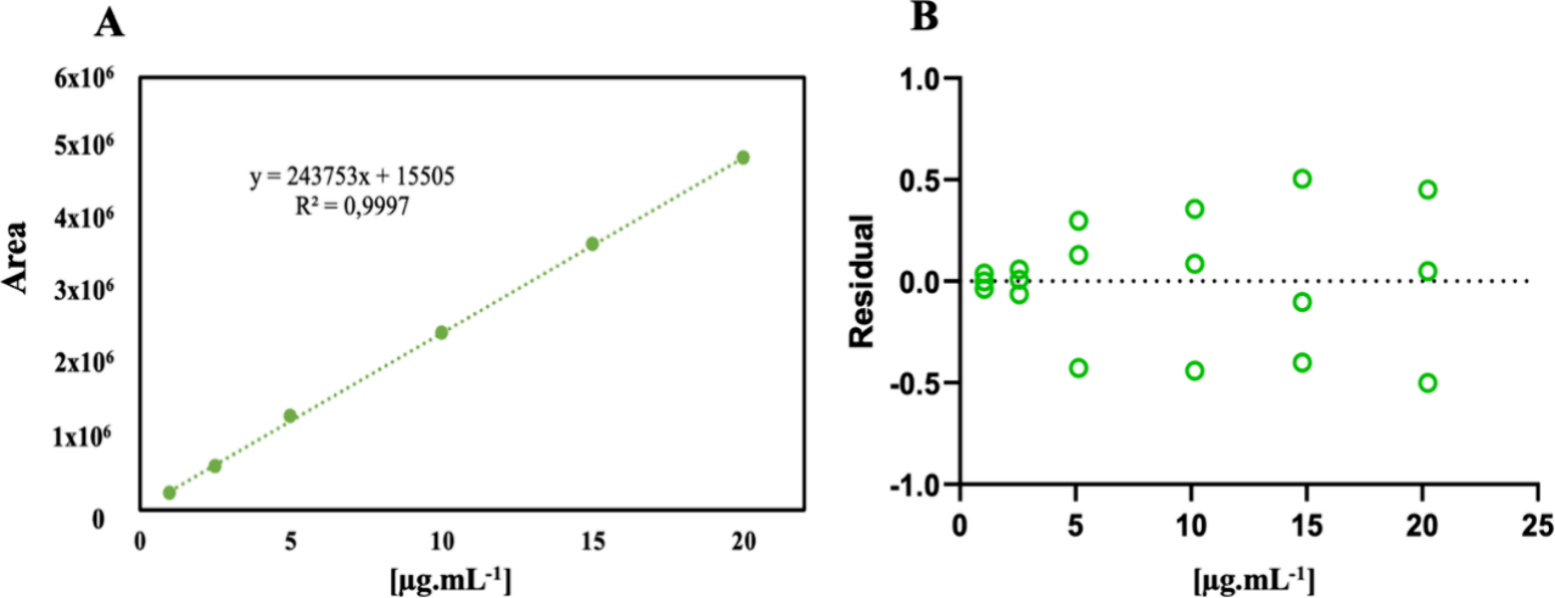
CS03 standard curve (A) and homoscedasticity of CS03 residues at concentrations ranging from 1 to 20 μg.mL^–1^ (B).

The linear regression of CS03 was derived from the standard curve (Figure 3A) using six concentrations (1–20 μg.mL^–1^). The resulting curve equation is y = 243753x + 15505, with a value of *r*^2^= 0.9997. The concentration of CS03 was determined by the equation [CS03] = (Abs +15505)/243753, where [CS03] represents the concentration of CS03 in μg.mL^–1^ and Abs indicates the peak area in μV s. The slope of the linear standard curve ranged from 234164 to 245552, significantly differing from zero.

After statistical analysis of the linearity data using ANOVA,^19^Figure 3B shows that the method presented homoscedasticity of the residues, thus indicating that the variations are the same as the normal distribution of the values identified in the linear regression model. The results of the F test showed that the comparison of variances was not significantly different with *F*_calculated_ = 1342 > F_tabulated_ = 3.11 with P value <0.0001.^30^ Thus, the proposed method is considered adequate to describe the linear regression in the range of CS03 concentrations from 1 to 20 μg.mL^–1^.

#### 3.2.3 LOD, LOQ, and Carryover

The LOD and LOQ values of this method were 0.21 and 0.66 μg.mL^–1^ respectively, being important data for the precision and accuracy of a method. The LOQ and LOD results were satisfactory, as they allow the analysis of CS03 in pharmaceutical formulations safely, showing that microgram scale concentrations can be reliably quantified by the HPLC-UV technique.^31^

**Figure 4:**
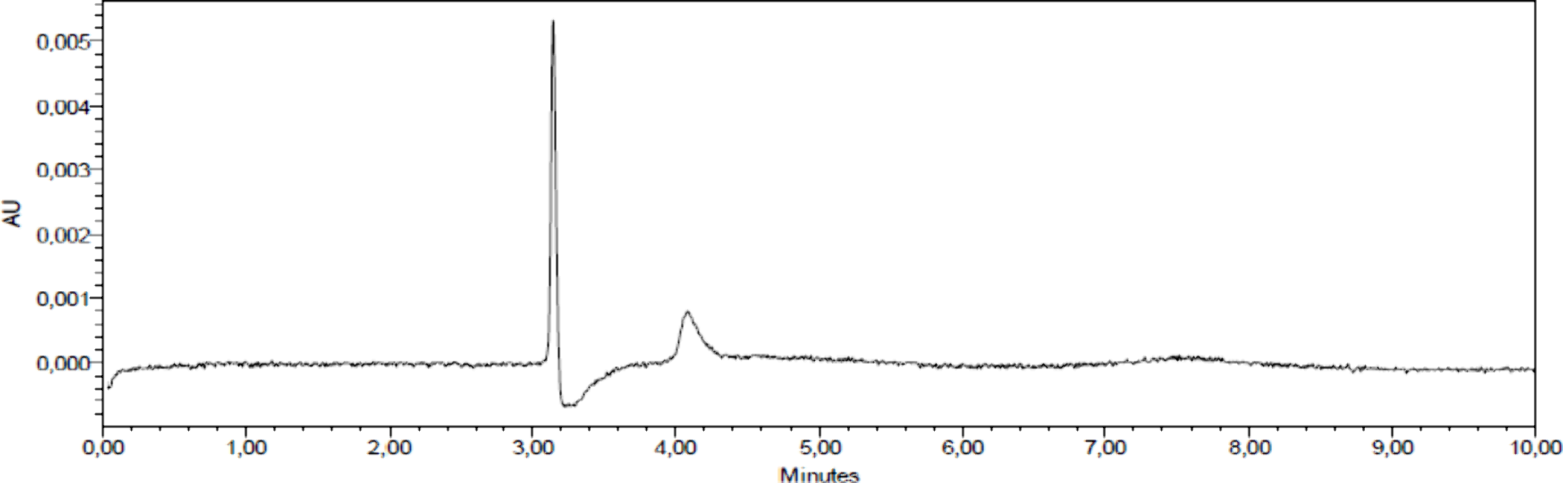
Chromatogram showing the presence of CS03 residue after elution at a concentration of 20 μg.mL-1.

As for the carry-over results, Figure 4 shows the presence of residue after the blank elution at the CS03 retention time. The presence of residues can influence the decrease of accuracy and precision in HPLC analyses, affecting subsequent analyses. The chemical characteristics of the analyte will contribute to the presence or absence of residues, as it is related to the affinity of the analyte for the stationary phase and/or the affinity with the eluent after the analyzes performed.^32,33^ The residue rate identified was 0.47%. This result is in line with the recommendations of the ICH^34^ guidelines which state that the blank sample response should be less than 20% of the peak LOQ response, which was found in this study to be 14.27%.

#### 3.2.4 Accuracy

In this study, the accuracy of the HPLC-UV method was evaluated using three concentration levels (1, 10, and 20 μg.mL^–1^) (Table 3), to identify how close the results obtained were to the theoretical concentration. The accuracy obtained between the concentrations ranged from 103.3 to 105.2%, in line with FDA^21^ recommendations, which are values ranging from 95 to 105%. The RSD values ranged from 1.69 to 3.58%. According to the results obtained, the validated method is considered accurate, following the recommendations of the ICH^34^ regarding the requirements for research into the quality of medicines.

**Table 3:** Accuracy in the Quantification of CS03 by the HPLC-UV Method

**CS03(μg.mL**^**–1**^**)**			
**Taken**	**Found ± SD**^a^	**Accuracy (%)**	**RSD**^a^**(%)**
1	1.05 ± 0.02	105.1	3.58
10	10.25 ± 0.17	102.6	2.47
20	20.65 ± 0.24	103.3	1.69

a SD: standard deviation, RSD: relative standard deviation.

#### 3.2.5 Precision

The evaluation of the method’s precision was performed from the quantification of CS03 in three concentrations (1, 10, and 20 μg.mL^–1^) by repeatability and intermediate precision on different days (interday), to which the data presented in Table 4 were evidenced. The values obtained for RSD after repeatability (intraday) ranged from 2.36 to 3.99%, while the interday data showed a variation of RSD between 0.75 to 3.51%. The data also meets the recommendations of the ICH^34^ guidelines, which state that the interday and intraday RSD precision should not exceed 15%.

**Table 4:** Results of the Study of Intraday and Interday Precision in the Quantification of CS03

**Intraday precision**			**Interday precision**		
Amount (μg.mL^–1^)			Amount (μg.mL^–1^)		
Taken	Found ± SD^a^	RSD^a^ (%)	Taken	Found ± SD^a^	RSD^a^ (%)
1	1.05 ± 0.02	3.38	1	1.03 ± 0.02	3.11
10	10.16 ± 0.29	3.99	10	10.12 ± 0.26	3.51
20	20.24 ± 0.33	2.36	20	20.60 ± 0.12	0.75

a SD: standard deviation, RSD: relative standard deviation.

**Figure 5:**
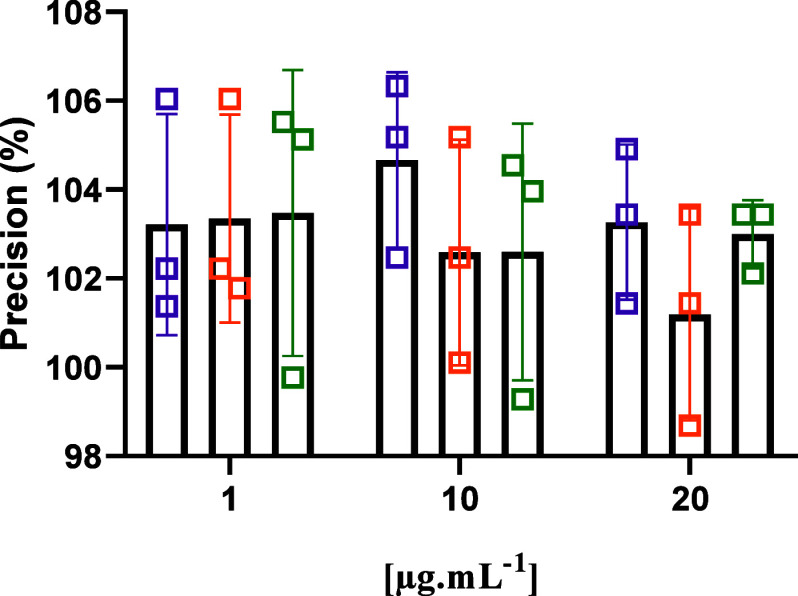
Intermediate precision of CS03 quantification by the HPLC-UV method: Analyst 1 (**purple** box open), Analyst 2 (**orange** box open), and Analyst 3 (**green** box open).

Precision RSD results show that there was a low number of significant changes between concentrations found with the method used. Furthermore, in Figure 5, it is possible to observe a low variation in terms of analyst change, with a coefficient of variation below 15% as recommended by the ICH.^34^ In view of the results obtained, the method showed low random errors in terms of repeatability and intermediate precisions.

#### 3.2.6 Robustness

The robustness of the method is related to the ability of the analytical method to resist small and deliberate variations in the analytical parameters.^35,36^ For robustness analyses, a concentration of 10 μg.mL^–1^ of CS03 was considered. The data obtained in this study (Table 5) showed that the variation in the mobile phase and flow ratio parameters directly impacted the CS03 concentration result. Therefore, it is reported that the parameters defined for the validation seem to be the most suitable for the quantification of CS03 and that variations in the flow parameters and proportion of the mobile phase may be critical for the quantification of this molecule and may present results that are not consistent with the real value of the CS03 concentration.

**Table 5:** Influence of Retention Time (Rt), Theoretical Concentration, and Content for CS03 Quantification

**CS03(μg.mL**^**–1**^**)**				
**Reference**	**Taken**	**Found**	**Content (%)**	**Rt (min)**
R1	10	11.84	118.42	4.48
R2	10	11.94	119.40	4.48
R3	10	11.66	116.62	4.48
R4	10	11.38	113.80	4.28
R5	10	8.75	87.59	4.10
R6	10	11.42	114.23	4.28
R7	10	8.86	88.65	4.13
R8	10	11.27	112.70	4.26
R9	10	8.72	87.28	4.18
R10	10	0.16	1.66	4.29
R11	10	0.17	1.70	4.30
R12	10	0.17	1.75	4.28
R13	10	10.24	102.47	4.10
R14	10	0.52	5.26	4.28
R15	10	10.01	100.15	4.10
R16	10	0.53	5.32	4.28
R17	10	10.38	103.84	4.10
R18	10	0.55	5.54	4.28
R19	10	0.45	4.53	4.66
R20	10	0.27	2.70	4.61
R21	10	0.30	3.02	4.64
R22	10	0.26	2.66	4.36
R23	10	0.28	2.84	4.18
R24	10	0.28	2.81	4.37
R25	10	0.28	2.84	4.22
R26	10	0.27	2.79	4.37
R27	10	0.27	2.75	4.21

### 3.3 Quantification of CS03 after Encapsulation in Nanoparticles

**Figure 6:**
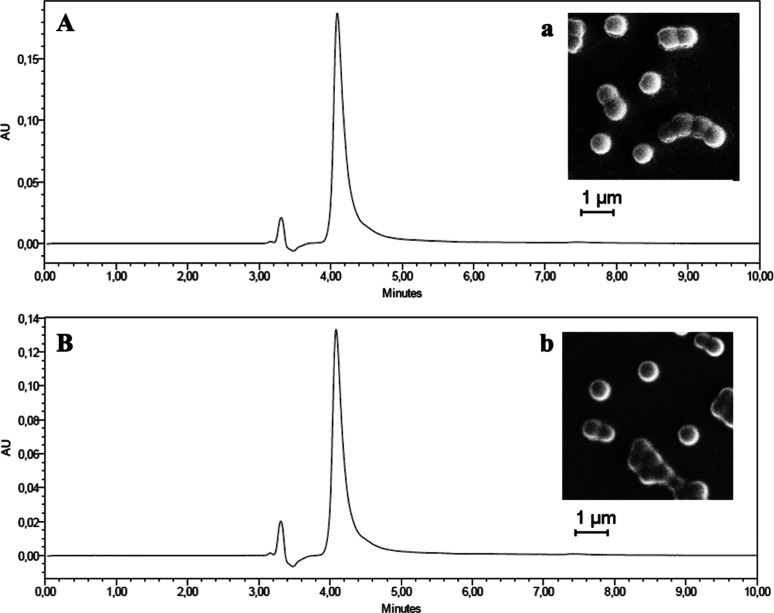
Chromatograms of CS03 after dissolving the dextran (A) and fucoidan (B) nanoparticles for the encapsulation rate study. The inset shows the shape of the dextran (a) and fucoidan (b) nanoparticles by scanning electron microscopy (SEM).

The development of dextran- or fucoidan-coated ETCA nanoparticles enabled the encapsulation of CS03.^4^ Despite the creation of the nanoparticles in an acidic medium (pH 2.5), CS03 remained stable, exhibiting a well-defined peak at a retention time of 4.21 min. These findings are consistent with those presented in Figure 2B, where the characteristics and retention time of the CS03 peak remain unchanged after encapsulation, indicating the molecule’s stability. The results illustrated in Figure 6 suggest that encapsulating CS03 in dextran- or fucoidan-coated nanoparticles can serve as a means to protect CS03 from degradation in acidic environments. Encapsulating the molecule in nanoparticles can also provide thermal protection, as well as photolytic protection, which could be studied in the future.

Depending on the coating of the nanoparticles, they may be an option for the protection of pH-sensitive drugs.^37,38^ According to the study by Date, Hanes, and Ensign,^39^ the encapsulation of drugs in nanoparticles improves their stability in environments with a wide range of pH, as in the case of the gastric-intestinal tract, in addition to increasing the solubility and bioavailability of the drug. The use of pH-resistant polysaccharides such as fucoidan, chitosan, and dextran, protects the drug from degradation and still allows the drug to be administered via oral, which is the preferred route, which has greater patient adherence to treatment.^18,40−42^

According to the study by Cavalcanti et al.,^17^ fucoidan-coated nanoparticles were shown to be resistant to gastric, intestinal, and blood pH. These results suggest that the encapsulation of CS03 in fucoidan and dextran nanoparticles was probably responsible for the protection of CS03 at acidic pH. The quantification of CS03 encapsulated in nanoparticles demonstrates that the method can be applied in *in vitro* assays to evaluate the controlled release of CS03, as well as in *in vivo* pharmacokinetic and bioavailability studies, after validation of the method by HPLC-UV for *in vivo* studies.

## 4 Conclusions

The development of a validation method for a promising drug candidate molecule is of paramount importance, as it facilitates the evaluation of the molecule’s behavior under various conditions and aids in the progression of a pharmaceutical product. This study delineates a validation method for the indole-thiazole derivative containing a p-nitro substituent (CS03), which has proven to be selective with appropriate linear regression, demonstrating data homoscedasticity, and exhibiting precision and accuracy. The concentrations derived from LOQ and LOD are suitable for pharmaceutical analysis, enabling the safe development and dosage of CS03 in pharmaceutical forms. Despite the satisfactory results achieved in the method validation, we identified that variations in the proportion of the mobile phase and flow rate are limiting factors of the method.

Furthermore, this method enabled us to identify the instability of CS03 at different pH levels, a factor that may restrict its future clinical application. However, our findings also suggest that encapsulating CS03 in nanoparticles coated with dextran or fucoidan appears to protect the molecule from degradation in acidic pH environments.

## Supporting Information Available

Combination of analytical parameters for the robustness study (Table S1) (PDF)

## References

[ref1] O’Dea A.; Sondergard C.; Sweeney P.; Arnatt C. K. A series of indole-thiazole derivatives act as GPER agonists and inhibit breast cancer cell growth. ACS Med. Chem. Lett. 2018, 9 (9), 901–906. 10.1021/acsmedchemlett.8b00212. 30258538 PMC6142053

[ref2] Alves J. E. F.; de Oliveira J. F.; de Lima Souza T. R. C.; de Moura R. O.; de Carvalho Júnior L. B.; Alves de Lima M. d. C.; de Almeida S. M. V. Novel índole-thiazole and índole-thiazolidinone derivatives as DNA groove binders. Int. J. Biol. Macromol. 2021, 170, 622–635. 10.1016/j.ijbiomac.2020.12.153. 33359805

[ref3] Soares J. C. S. Síntese, caracterização estrutural e avaliação de potencial atividade antitumoral de novos derivados indol-tiazólicos. Dissertation (Master), 116 p. Federal University of Pernambuco, Recife-PE 2020.

[ref4] Soares J. C. S.; Cavalcanti I. D. L.; Cruz-Filho I. J.; Lira Nogueira M. C. B.; Lima M. C. A. Antiproliferative activity in metastatic breast cancer cells of nanoparticles containing a novel indole-thiazole derivative. Colloids Surf., A 2024, 700, 134794 10.1016/j.colsurfa.2024.134794.

[ref5] Soares J. C. S. Síntese, caracterização estrutural e avaliação da atividade antiproliferativa de novos derivados indol-tiazólicos livres e encapsulados em nanopartículas poliméricas. Thesis (Doctorate), 187 p. Federal University of Pernambuco, Recife-PE 2023.

[ref6] Rao T. N. Validation of analytical methods. Intechopen 2018.

[ref7] Tavares C. A.; Xavier-Júnior F. H.; Pessoa O. D. L.; Ximenes R. M.; Santos-Magalhães N. S. Validation of an HPLC-UV method for quantifying oncocalyxone A in different media and nanopcapsules. Chromatographia 2019, 82, 809–818. 10.1007/s10337-019-03716-x.

[ref8] Gómez R. R.; Valencia P. R. In vitro-in vivo pharmacokinetic correlation model for quality assurance of antiretroviral drugs. Colomb. Méd. 2015, 46 (3), 109–116. 10.25100/cm.v46i3.1650. PMC4640432 26600625

[ref9] Mennickent S.; Diego M. Analytical method validation as the first step in drug quality control. In Quality Management and Quality Control Intechopen 2019.

[ref10] Fink C.; Sun D.; Wagner K.; Schneider M.; Bauer H.; Dolgos H.; Mader K.; Peters S. A. Evaluating the role of solubility in oral absorption of poorly water-soluble drugs using physiologically-based pharmacokinetic modeling. Clinical Pharmacology and Therapeutics 2020, 107 (3), 650–661. 10.1002/cpt.1672. 31608434 PMC7158207

[ref11] Mazzucco E.; Gosetti F.; Bobba M.; Marengo E.; Robotti E.; Gennaro M. C. High-performance liquid chromatography-ultraviolet detection method for the simultaneous determination of typical biogenic amines and precursor amino acids. Applications in food chemistry. J. Agric. Food Chem. 2010, 58 (1), 127–134. 10.1021/jf9030053. 19928988

[ref12] Michels L. R.; Fachel F. N. S.; Azambuja J. H.; Gelsleichter N. E.; Braganhol E.; Teixeira H. F. HPLC-UV method for Temozolomide determination in complex biological matrices: application for *in vitro*, *ex vivo* and *in vivo* studies. Biomed. Chromatogr. 2019, 33 (10), e4615 10.1002/bmc.4615. 31166608

[ref13] Brugnera M.; Vicario-de-la-Torre M.; Andrés-Guerrero V.; Bravo-Osuna I.; Molina-Martínez I. T.; Herrero-Vanrell R. Validation of a rapid and easy-to-apply method to simultaneously quantify co-loaded dexamethasone and melatonin PLGA microspheres by HPLC-UV: encapsulation efficiency and in vitro release. Pharmaceutics 2022, 14, 288 10.3390/pharmaceutics14020288. 35214021 PMC8878730

[ref14] Martins L. G.; Khalil N. M.; Mainardes R. M. Application of a validated HPLC-PDA method for the determination of melatonin content and its release from poly(lactic acid) nanoparticles. Journal of Pharmaceutical Analysis 2017, 7 (6), 388–393. 10.1016/j.jpha.2017.05.007. 29404064 PMC5790749

[ref15] Tome T.; Zigart N.; Casar Z.; Obreza A. Development and optimization of liquid chromatography analytical methods by using AQbD principles: overview and recent advances. Org. Process Res. Dev. 2019, 23 (9), 1784–1802. 10.1021/acs.oprd.9b00238.

[ref16] Vincent U.; Serano F.; von Holst C. Validation of a multi-analyte HPLC method for the determination of carotenoids used as feed additives in fish and poultry feed: results of an interlaboratory study. Food Additives & Contaminants: Part A 2021, 38 (3), 396–408. 10.1080/19440049.2020.1869325. 33481680

[ref17] Cavalcanti I. D. L.; Ximenes R. M.; Pessoa O. D. L.; Magalhães N. S. S.; de Britto Lira-Nogueira M. C. Fucoidan-coated PIBCA nanoparticles containing oncocalyxone A: Activity against metastatic breast cancer cells. J. Drug Delivery Sci. Technol. 2021, 65, 102698 10.1016/j.jddst.2021.102698.

[ref18] Cavalcanti I. D. L.; de Aguiar Silva A. T.; da Silva Maciel V.; Tavares J. L.; Santos-Magalhães N. S.; de Britto Lira-Nogueira M. C. Are poly(isobutylcyanoacrylate) nanoparticles a promising nanosystem?. J. Nanopart. Res. 2024, 26, 118 10.1007/s11051-024-06031-1.

[ref19] International Conference on Harmonization (ICH) Validation of analytical procedures: text and methodology Q2 (R1), November 2005. Available on: https://www.ich.org/fileadmin/Public_Web_Site/ICH_Products/Guidelines/Quality/Q2_R1/Step4/Q2_R1__Guideline.pdf 2005. Accessed on: 26 Aug 2022.

[ref20] Food and Drug Administration (FDA) Analytical procedures and methods validation for drugs and biologics: guidance for industry, 2015. Available on: https://www.fda.gov/downloads/drugs/guidances/ucm386366.pdf 2015. Accessed on: 26 Aug 2022.

[ref21] Food and Drug Administration (FDA) (2020) Methods, Method Verification and Validation, 2020. Available on: https://www.fda.gov/media/73920/download. Accessed on: 28 Aug 2022.

[ref22] Agência Nacional de Vigilância Sanitária (ANVISA) RDC N°166–24 July 2017. Available on: http://portal.anvisa.gov.br/documents/10181/2721567/RDC_166_2017_COMP.pdf/d5fb92b3-6c6b-4130-8670-4e3263763401. Accessed on: 26 Aug 2022.

[ref23] Lira M. C. B.; Santos-Magalhães N. S.; Nicolas V.; Marsaud V.; Silva M. P. C.; Ponchel G.; Vauthier C. Cytotoxicity and cellular uptake of newly synthesized fucoidan-coated nanoparticles. Eur. J. Pharm. Biopharm. 2011, 79 (1), 162–170. 10.1016/j.ejpb.2011.02.013. 21349331

[ref24] Czyrski A.; Sznura J. The application of Box-Behnken-Design in the optimization of HPLC separation of fluoroquinolones. Sci. Rep. 2019, 9, 19458 10.1038/s41598-019-55761-z. 31857613 PMC6923357

[ref25] Piekarski M.; Dołhań A.; Cielecka-Piontek J.; Zalewski P.; Kycler W.; Kaczmarek A.; Firlej A.; Oszczapowicz I.; Jelińska A. The influence of pH and temperature on the stability of *N*-[(Piperidine)methylene]daunorubicin hydrochloride and a comparison of the stability of daunorubicin and its four new amidine derivatives in aqueous solutions. Sci. World J. 2014, 2014, 803789 10.1155/2014/803789. PMC3933308 24688433

[ref26] Jangid A. K.; Pooja D.; Kulhari H. Determination of solubility, stability and degradation kinetics of morin hydrate in physiological solutions. RSC Adv. 2018, 8, 28836–28842. 10.1039/C8RA04139C. 35548393 PMC9084450

[ref27] Mathew M.; Gupta V. D.; Bailey R. E. Stability of omeprazole solutions at various ph values as determined by high-performance liquid chromatography. Drug Dev. Ind. Pharm. 1995, 21 (8), 965–971. 10.3109/03639049509026660.

[ref28] Conner J.; Wuchterl D.; Lopez M.; Minshall B.; Prusti R.; Boclair D.; Peterson J.; Allen C. Chapter 26 – The Biomanufacturing of Biotechnology Products. Biotechnology Entrepreneurship 2014, 351–385. 10.1016/B978-0-12-404730-3.00026-9.

[ref29] Akula P.; Lakshmi P. K. Effect of pH on weakly acidic and basic model drugs and determination of their *ex vivo* transdermal permeation routes. Braz. J. Pharm. Sci. 2018, 54 (2), e00070 10.1590/s2175-97902018000200070.

[ref30] Mahbobi M.; Tieman T. K. F-test and one-way ANOVA. In Introductory business statistics with interactive spreadsheets-1st Canadian Edition 2015.

[ref31] Armbruster D. A.; Pry T. Limit of blank, limit of detection and limit of quantification. Clin. Biochem. Rev. 2008, 29 (Suppl 1), S49–S52. 18852857 PMC2556583

[ref32] Buszewski B.; Noga S. Hydrophilic interaction liquid chromatography (HILIC)- a powerful separation technique. Anal. Bioanal. Chem. 2012, 402 (1), 231–247. 10.1007/s00216-011-5308-5. 21879300 PMC3249561

[ref33] Moein M. M.; El Beqqali A.; Abdel-Rehim M. Bioanalytical method development and validation: critical concepts and strategies. Journal of Chromatography B 2017, 1043, 3–11. 10.1016/j.jchromb.2016.09.028. 27720355

[ref34] International Conference on Harmonization (ICH), ICH guideline M10 on bioanalytical method validation and study sample analysis. European Medicines Agency, 2020 July 2022. Available on: https://www.ema.europa.eu/en/documents/scientific-guideline/ich-guideline-m10-bioanalytical-method-validation-step-5_en.pdf. Accessed on: 27 Aug 2022.

[ref35] César I. C.; Pianetti G. A. Robustness evaluation of the chromatographic method for the quantification of lumefantrine using youden’s test. Brazilian Journal of Pharmaceutical Sciences 2009, 45 (2), 235–240. 10.1590/S1984-82502009000200007.

[ref36] Ferreira S. L. C.; Caires A. O.; Borges T. D. S.; Lima A. M. D. S.; Silva L. O. B.; dos Santos W. N. L. Robustness evaluation in analytical methods optimized using experimental designs. Microchem. J. 2017, 131, 163–169. 10.1016/j.microc.2016.12.004.

[ref37] Chenthamara D.; Subramaniam S.; Ramakrishnan S. G.; Krishnaswamy S.; Essa M. M.; Lin F. H.; Qoronfleh M. W. Therapeutic efficacy of nanoparticles and routes of administration. Biomater. Res. 2019, 23, 20 10.1186/s40824-019-0166-x. 31832232 PMC6869321

[ref38] Mitchell M. J.; Billingsley M. M.; Haley R. M.; Wechsler M. E.; Peppas N. A.; Langer R. Engineering precision nanoparticles for drug delivery. Nat. Rev. Drug Discovery 2021, 20, 101–124. 10.1038/s41573-020-0090-8. 33277608 PMC7717100

[ref39] Date A. A.; Hanes J.; Ensign L. M. Nanoparticles for oral delivery: design, evaluation and state-of-the-art. J. Controlled Release 2016, 240, 504–526. 10.1016/j.jconrel.2016.06.016. PMC5064878 27292178

[ref40] Barbosa A. I.; Costa Lima S. A.; Reis S. Application of pH-responsive fucoidan/chitosan nanoparticles to improve oral quercetin delivery. Molecules 2019, 24 (2), 346 10.3390/molecules24020346. 30669398 PMC6359289

[ref41] Palanikumar L.; Al-Hosani S.; Kalmouni M.; Nguyen V. P.; Ali L.; Pasricha R.; Barrera F. N.; Magzoub M. pH-responsive high stability polymeric nanoparticles for targeted delivery of anticancer therapeutics. Commun. Biol. 2020, 3, 95 10.1038/s42003-020-0817-4. 32127636 PMC7054360

[ref42] Yuba E.; Uesugi S.; Miyazaki M.; Kado Y.; Harada A.; Kono K. Development of pH-sensitive dextran derivatives with strong adjuvant function and their application to antigen delivery. Membranes 2017, 7 (3), 41 10.3390/membranes7030041. 28777336 PMC5618126

