# Supplementary material for: Novel Indole–Thiazole Derivative Containing a p-Nitro Substituent (CS03): Validation of an HPLC-UV Quantification Method
Source: ACS Omega. 2025 Apr 9;10(15):15697–705. doi: 10.1021/acsomega.5c01148 (PMC12019425; doi:10.1021/acsomega.5c01148)
Supplement: Supplementary file 1 — ao5c01148_si_001.pdf [file ao5c01148_si_001.pdf]

Novel indole-thiazole derivative containing a p-nitro substituent (CS03): Validation of an HPLC-UV quantification method

José Cleberson Santos Soares[1*], Iago Dillion Lima Cavalcanti[2,3*], Iranildo José da Cruz-Filho[1], Mariane Cajubá de Britto Lira Nogueira[2,3], Maria do Carmo Alves de Lima[1]

[1] Laboratório de Química e Inovação Terapêutica (LQIT), Universidade Federal de Pernambuco (UFPE), Recife, Brazil.

[2] Instituto Keizo-Asami (iLIKA), Universidade Federal de Pernambuco (UFPE), Recife, Brazil;

[3] Laboratório de Nanotecnologia, Biotecnologia e Cultura de Células, Centro Acadêmico de Vitória, Universidade Federal de Pernambuco (CAV/UFPE), Recife, Brazil.

*Corresponding authors:

José Cleberson Santos Soares / Iago Dillion Lima Cavalcanti

Federal University of Pernambuco

Av. Prof. Moraes Rego, 1235, Cidade Universitária, 50670-901, Recife-PE, Brazil

E-mail: cleberson.soares@ufpe.br /iago.dillion@ufpe.br

Supplementary material

Table S1. Combination of analytical parameters for the robustness study.

**Factorial combination**	**Analytical parameters**				
	Concentration of acetonitrile in the mobile phase	Concentration of acidified water in the mobile phase	Column temperature	Mobile phase flow rate	Reference
1	AA	BB	CC	DD	R1
2	AA	BB	Cc	DD	R2
3	AA	BB	cc	DD	R3
4	AA	BB	CC	Dd	R4
5	AA	BB	CC	dd	R5
6	AA	BB	cc	Dd	R6
7	AA	BB	cc	dd	R7
8	AA	BB	Cc	Dd	R8
9	AA	BB	Cc	dd	R9
10	Aa	Bb	CC	DD	R10
11	Aa	Bb	Cc	DD	R11
12	Aa	Bb	cc	DD	R12
13	Aa	Bb	CC	Dd	R13
14	Aa	Bb	CC	dd	R14
15	Aa	Bb	cc	Dd	R15
16	Aa	Bb	cc	dd	R16
17	Aa	Bb	Cc	Dd	R17
18	Aa	Bb	Cc	dd	R18
19	aa	bb	CC	DD	R19
20	aa	bb	Cc	DD	R20
21	aa	bb	cc	DD	R21
22	aa	bb	CC	Dd	R22
23	aa	bb	CC	dd	R23
24	aa	bb	cc	Dd	R24
25	aa	bb	cc	dd	R25
26	aa	bb	Cc	Dd	R26
27	aa	bb	Cc	dd	R27
